# MicroRNA expression profile in exosome discriminates extremely severe infections from mild infections for hand, foot and mouth disease

**DOI:** 10.1186/1471-2334-14-506

**Published:** 2014-09-17

**Authors:** Hong-Ling Jia, Chun-Hui He, Zhuo-Ya Wang, Yu-Fen Xu, Gen-Quan Yin, Li-Jia Mao, Chao-Wu Liu, Li Deng

**Affiliations:** Guangzhou Women and Children’s Medical Center, Guangzhou, 510120 Guangdong, China; Key Laboratory of Functional Protein Research of Guangdong Higher Education Institutes, Institute of Life and Health Engineering, College of Life Science and Technology, Jinan University, Guangzhou, 510632 Guangdong China; Guangdong Institute of Microbiology/State Key Laboratory of Applied Microbiology Southern China/Guangdong Provincial Key Laboratory of Microbial Culture Collection and Application, Guangzhou, 510070 Guangdong China; Guangdong Provincial Key Laboratory of Pharmaceutical Bioactive Substances, School of Basic Courses, Guangdong Pharmaceutical University, Guangzhou, 510006 Guangdong China

**Keywords:** Exosomal microRNA Profile, HFMD, Diagnosis, Biomarker

## Abstract

**Background:**

Changes of miRNAs in exosome have been reported in different disease diagnosis and provided as potential biomarkers. In this study, we compared microRNA profile in exosomes in 5 MHFMD and 5 ESHFMD as well as in 5 healthy children.

**Methods:**

Different expression of miRNAs in exosomes across all the three groups were screened using miRNA microarray method. Further validated test was conducted through quantitative real-time PCR assays with 54 exosome samples (18 ESHFMD, 18 MHFMD, and 18 healthy control). The judgment accuracy was then estimated by the receiver operating characteristic (ROC) curve analysis; and the specificity and sensitivity were evaluated by the multiple logistic regression analysis.

**Results:**

There were 11 different miRNAs in exosomes of MHFMD and ESHFMD compared to healthy children, of which 4 were up-regulated and 7 were down-regulated. Further validation indicated that the 4 significant differentially expressed candidate miRNAs (miR-671-5p, miR-16-5p, miR-150-3p, and miR-4281) in exosome showed the same changes as in the microarray analysis, and the expression level of three miRNAs (miR-671-5p, miR-16-5p, and miR-150-3p) were significantly different between MHFMD or ESHFMD and the healthy controls. The accuracy of the test results were high with the under curve (AUC) value range from 0.79 to 1.00. They also provided a specificity of 72%-100% and a sensitivity of 78%-100%, which possessed ability to discriminate ESHFMD from MHFMD with the AUC value of 0.76-0.82.

**Conclusions:**

This study indicated that the exosomal miRNA from patients with different condition of HFMD express unique miRNA profiles. Exosomal miRNA expression profiles may provide supplemental biomarkers for diagnosing and subtyping HFMD infections.

**Electronic supplementary material:**

The online version of this article (doi:10.1186/1471-2334-14-506) contains supplementary material, which is available to authorized users.

## Background

Hand, foot, and mouth disease (HFMD) is a common acute viral illness which has been epidemic worldwide [1–3]. The two major causative agents are known as human enterovirus 71 (EV71) [4] and coxsackievirus A16 (CVA16), which accounting for more than 70% of cases in recent outbreaks [5]. Moreover, the effective and reliable tool for diagnosing of HFMD is not available [6, 7].

The extremely severe HFMD (ESHFMD) mainly caused by EV71 with severe neurologic clinical symptoms and significant fatalities [8–10], that had caused serious public health concerns. Many children with extremely severe HFMD were died before making a definite diagnosis. Thus, a rapid and reliable diagnostic method is essential for appropriate treatment and prophylaxis.

Exosome represent a specific subtype of secreted membrane vesicles that are approximately 30–100 nm in size and are formed inside the secreting cells in endosomal compartments called multivesicular bodies [11, 12]. Secreted vesicles play an important role in normal physiological processes, development, and conditions such as viral infection [13–15] and are a possible source or pool of novel biomarkers of many diseases [16–18]. Exosome contain proteins, miRNAs, and mRNAs, and the exosomal lipid bilayer protects this genetic information from degradation. Moreover, miRNAs can be transferred by an exosomal route and further exert gene silencing in recipient cells [19, 20], where they play an essential regulatory role during development, with their levels changing in different cell types and at different developmental stages [21, 22]. Although the mechanistic basis for alterations in miRNA, especially in the context of cellular malfunction, is not well understood, such alterations play a pivotal role in pathological processes and have recently been proposed as biomarkers for brain neoplasms, degenerative diseases, autism, and schizophrenia [23–25]. Changes in exosomal miRNAs have been reported in patients diagnosed with Alzheimer’s disease (AD), and miRNAs have been shown to provide diagnostic biomarkers [26].

Here, we employed microarray methods to compare the miRNAs of exosome from serum samples collected from normal children and patients with mild HFMD (MHFMD) and extremely severe HFMD (ESHFMD). We focused on the miRNA profile of exosome and confirmed whether these changes could be used to discrimination of specific condition for ESHFMD and MHFMD.

## Methods

### Serum sample preparation

Ethical approval was obtained for human sample collection from the Ethics Committees at Guangzhou Women and Children’s Medical Center, and written informed consent was obtained from all guardians. Blood samples from five MHFMD and five ESHFMD children diagnosed according to the *Hand Foot and Mouth Disease Prevention Control Guide* (2008 edition) issued by the Ministry of Health of China (http://www.moh.gov.cn/publicfiles/business/htmlfiles/mohbgt/s9511/200805/34775.htm) were randomly collected for 2-DE, and clinical symptoms and laboratory testing (EV71 nucleic acid detection kit) confirmed that EV71 infection caused HFMD in all these cases. In addition to meeting the above criteria, ESHFMD patients all had encephalitis and pulmonary haemorrhage, required mechanical ventilation, and had other clinical symptoms. They were confirmed to have no other disease after a systematic check in the hospital. Five blood samples from healthy children were collected as controls. To validate the miRNA microarray results, we randomly collected blood samples of 18 ESHFMD patients and 18 MHFMD patients according to the diagnostic guidelines described above and subjected the samples to real-time quantitative RT-PCR. Another 18 blood samples from healthy children were collected as controls. Blood samples were separated by centrifugation at 1,000 × *g* for 10 min. Serum aliquots were collected and stored at -80°C. The serum obtained was further processed for exosome isolation.

### ExoQuick precipitation of serum exosome

We isolated exosome from the sera of all participants by using ExoQuick precipitation (System Biosciences Inc, Mountain View, CA) following the manufacturer’s instructions [27, 28].

### Exosome characterization

#### Transmission electron microscopy (TEM)

The exosome extraction reagent was used to precipitate the exosome from serum, which were then centrifuged at 1,500 × *g* for 10 min at 4°C to remove the supernatant. The exosome pellet was resuspended in 10 mM PBS in four times the volume of serum. A copper mesh was placed on a clean wax plate, and 100 μl of the exosome suspension was added. After 4 min, the copper mesh was removed and placed in 2% phosphotungstic acid for 5 min. The mesh was laid on the filter paper for air-drying, and TEM was used to observe the morphological features of the exosome.

#### Western blot analysis

The exosome pellet was dissolved in the protein lysis buffer, and the protein concentration was determined using a Bradford protein assay kit (Bio-Rad, USA). Samples were separated on a 1D SDS-PAGE gel before transfer to a PVDF membrane. The membrane was incubated with the TSG101, CD63, CD9, HSP90α and Flotillin primary antibodies at 4°C overnight, followed by incubation with the corresponding secondary antibodies at room temperature for 1 h. Specific protein bands were visualized using the SuperSignal chemiluminescence system (ECL, Pierce, USA) and imaged by autoradiography.

### RNA extraction from exosome

RNA was extracted from the exosome pellets using TRIZOL reagent according to the manufacturer’s protocol. Briefly, 1.0 ml of TRIZOL reagent and 200 μl of chloroform were added to the sample, and the mixture was vortexed for 60 s and allowed to stand at 25°C for 5 min. After the mixture was centrifuged at 10,000 × *g* for 10 min at 4°C, the supernatant was transferred to a fresh tube and 500 μl of isopropanol was added. After incubation at -20°C overnight, the mixture was centrifuged at 10,000 × *g* for 10 min at 4°C to remove the supernatant, and the RNA pellet was washed with 75% ethanol. After ethanol removal by centrifugation at 10,000 × *g* for 10 min at 4°C, the RNA was air-dried for 5 min and then dissolved in 20 μl of RNase-free water. The purity of the isolated RNA was determined according to the OD260/280 using a Nanodrop ND-1000 system (Thermo Fisher Scientific).

### Microarray analysis

Pooled exosome of serum from five healthy children, five MHFMD and five ESHFMD patients were used for miRNA microarray analysis. Total miRNA from these three pooled samples was extracted as described above. Microarray hybridization, data generation, and normalization were performed by the Shanghai Biochip Corp. following standard Agilent protocols. Human miRNA microarrays from Agilent Technologies, which contain probes for 1,887 human miRNAs from the Sanger database v.18.0, were used in this study. Visualization of microarray data was performed using MeV 4.6 software (MultiExperiment Viewer; http://www.tm4.org/mev.html). The microarray data described herein have been deposited in the National Center for Biotechnology Information Gene Expression Omnibus (http://www.ncbi.nlm.nih.gov/geo/), with accession number GSE52780. A miRNA was designated as overexpressed if expression in one of the pooled samples was >1.5-fold higher than that in another sample. In cases where overexpression was determined for a differentially expressed miRNA, miRNA with the maximum intensity over 4 (log2 transformed intensity) in at least one of the paired samples was considered.

### Validation of real-time quantitative PCR

For testing of candidate miRNAs identified by microarrays, real-time quantitative PCR (qRT-PCR) was performed using the Power SYBR Green PCR Master Mix (Applied Biosystems) in an ABI 7500 Real-Time PCR System (Applied Biosystems). The assays were performed on 54 samples (18 Control, 18MHFMD and 18 ESHFMD) for four candidates (miR-16-5p, miR-15-3p, mir-4281, and miR-671) that met the defined criteria. Each reaction was performed in a 20 μl volume system containing 5 μl of cDNA, 0.5 μl of each primer, 10 μl of Power SYBR Green PCR Master Mix, and 4.0 μl of RNase-free water. The PCR program consisted of denaturation at 95°C for 2 min, followed by 40 cycles each of denaturation for 15 s at 95°C and annealing and extension for 30 s at 60°C. The miR-642a-3p expression level was used as a stable endogenous control for normalization. All assays were conducted in triplicate. miRNAs that showed cycle threshold values above 35 in one of the 54 samples were excluded from additional statistical analysis.

### Target prediction and enrichment information

The target genes of the candidate miRNAs were predicted by TargetScan prediction software (http://www.targetscan.org/). The Gene Ontology (GO) and Kyoto Encyclopedia of Genes and Genomes (KEGG) database analyses were conducted using a DAVID online analysis tool (http://david.abcc.ncifcrf.gov/), in which we focused on the Gene Ontology (GO) biological processes feature. For each analysis, we used *P* < 0.05 as a cut-off.

### Statistical analysis

For the qRT-PCR data, relative miRNA expression levels were calculated by the comparative 2^-△△Ct^ method as described previously [29]. Statistical significance was determined using Student’s *t*-test. *P* < 0.05 was considered statistically significant. Receiver operating characteristic (ROC) curves were constructed to determine the specificity and sensitivity of individual miRNAs as surrogate biomarkers. Area under the ROC curve (AUC) was used as an accuracy index for evaluating the diagnostic performance of the selected miRNA panel. MedCalc (version 10.4.7.0; MedCalc, Mariakerke, Belgium) software was used to perform the ROC analysis.

## Results

### Exosome isolation and validation

**Figure 1:**
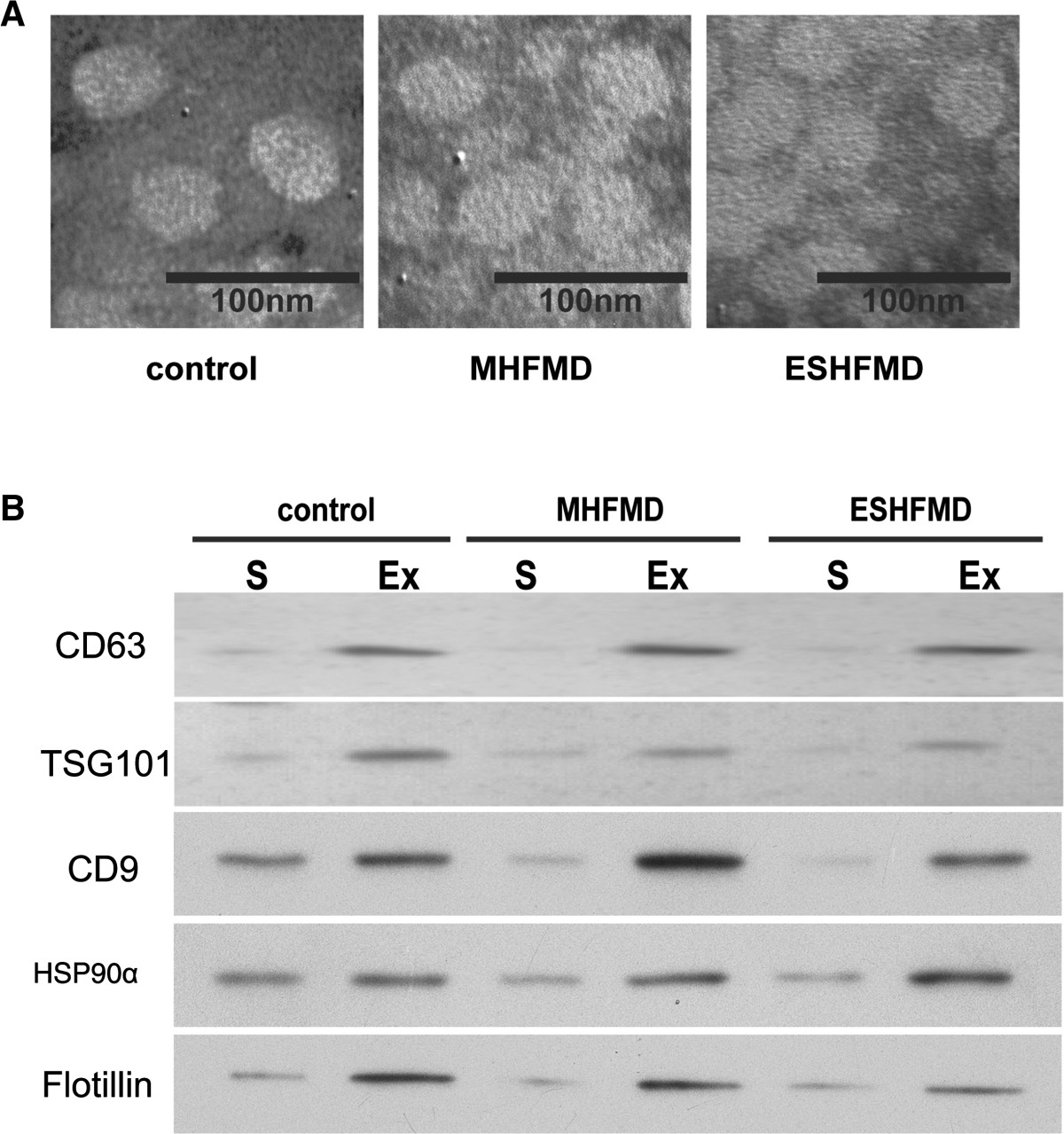
**Characterization of exosomes in serum samples from healthy children and MHFMD and ESHFMD patients by transmission electron microscopy and western blotting. (A)** Morphological characterization by transmission electron microscopy. **(B)** Molecular characterization by western blot analysis. Protein extracts prepared from serum (S) or exosomes (Ex) were assessed using antibodies against exosomal protein markers (TSG101, CD63, CD9, HSP90α and Flotillin).

Microvesicles isolated from sera of controls and MHFMD and ESHFMD patients were assessed by TEM and western blotting. TEM showed spherical structures approximately 30–100 nm in diameter, consistent with previously reported characteristics of exosomes (Figure 1A). We further confirmed that these microvesicles were exosome by performing western blot analysis on lysates using antibodies against five commonly used exosomal markers, TSG101, CD63, CD9, HSP90α and Flotillin. Levels of TSG101, CD63, CD9, HSP90α and Flotillin were strikingly higher in the microvesicle fraction than in serum (Figure 1B). These results confirmed the identification and characterization of isolated microvesicles as exosomes.

### Global exosome miRNA profiling from microarray analysis

**Figure 2:**
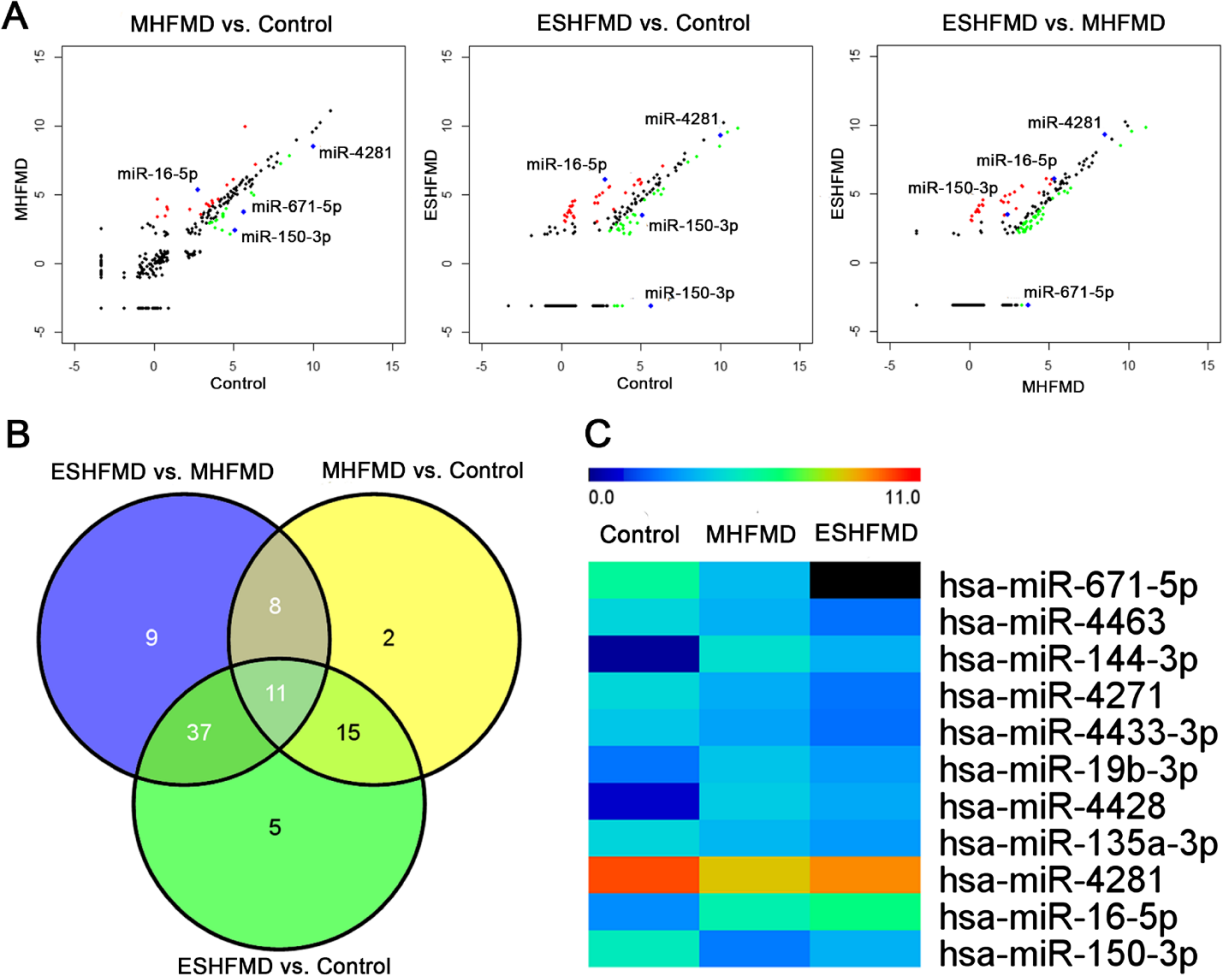
**Microarray analysis of HFMD. (A)** Scatter plot of miRNA expression determined by miRNA microarray analysis. The expression profile of 1,887 miRNAs on a log2 scale in HFMD and in the control group is plotted. Red and green dots represent the number of miRNAs that were significantly (*P* < 0.05 and 1.5 fold-change cut-off) up-regulated and down-regulated, respectively, in HFMD, and black dots represent a lack of differential expression. (a) Comparison between normal controls and MHFMD samples, (b) comparison between normal controls and ESHFMD samples, and (c) comparison between MHFMD and ESHFMD samples. **(B)** Venn diagram illustrates the overlapping results of the differentially expressed miRNAs in MHFMD vs. control, ESHFMD vs. control, and MHFMD vs. ESHFMD comparisons. Circles include numbers of up-regulated or down-regulated human miRNAs of each pair-wise comparison. The 11 miRNAs in the centre of the Venn diagram represent miRNAs that are differentially expressed in all group comparisons. **(C)** Heat map analysis of differentially regulated miRNAs among ESHFMD, MHFMD, and control serum samples according to Venn diagram analysis. Heat map colours represent relative miRNA expression as indicated in the colour key. Red represents high expression, whereas green represents low expression.

To screen for candidate exosomal miRNAs from MHFMD and ESHFMD patient serum samples, miRNA microarrays were used to evaluate the three groups (MHFMD, ESHFMD, and control). The microarray results identified various miRNAs that were differentially regulated in the exosome of MHFMD and ESHFMD samples relative to healthy controls, and a scatter plot was generated (Figure 2A). Subsequently, we conducted pairwise comparison of the results of the scatter plot charts and found 36 miRNAs with significantly different expression between MHFMD and the control, 68 miRNAs differentially expressed between ESHFMD and the control, and 65 miRNAs differentially expressed between ESHFMD and MHFMD. Eleven miRNAs, that are miR-671-5p, miR-4463, miR-144-3p, miR-4271, miR-4433-3p, miR-19b-3p, miR-4428, miR-135a-3p, miR-4281, miR-16-5p, and miR-150-3p, showed significantly different expression across all three groups (Figure 2B), indicating a clear distinction between the MHFMD, ESHFMD, and control (Figure 2C). Four of these miRNAs were up-regulated and seven were down-regulated in MHFMD and ESHFMD serum samples compared to the control. miR-671-5p was only appeared in healthy children and MHFMD, and was almost undetectable in ESHFMD patients. These data can provide a valuable repertoire to discover miRNA-based biomarkers for distinguishing ESHFMD from MHFMD.

### qRT-PCR verification of miRNA expression

**Figure 3:**
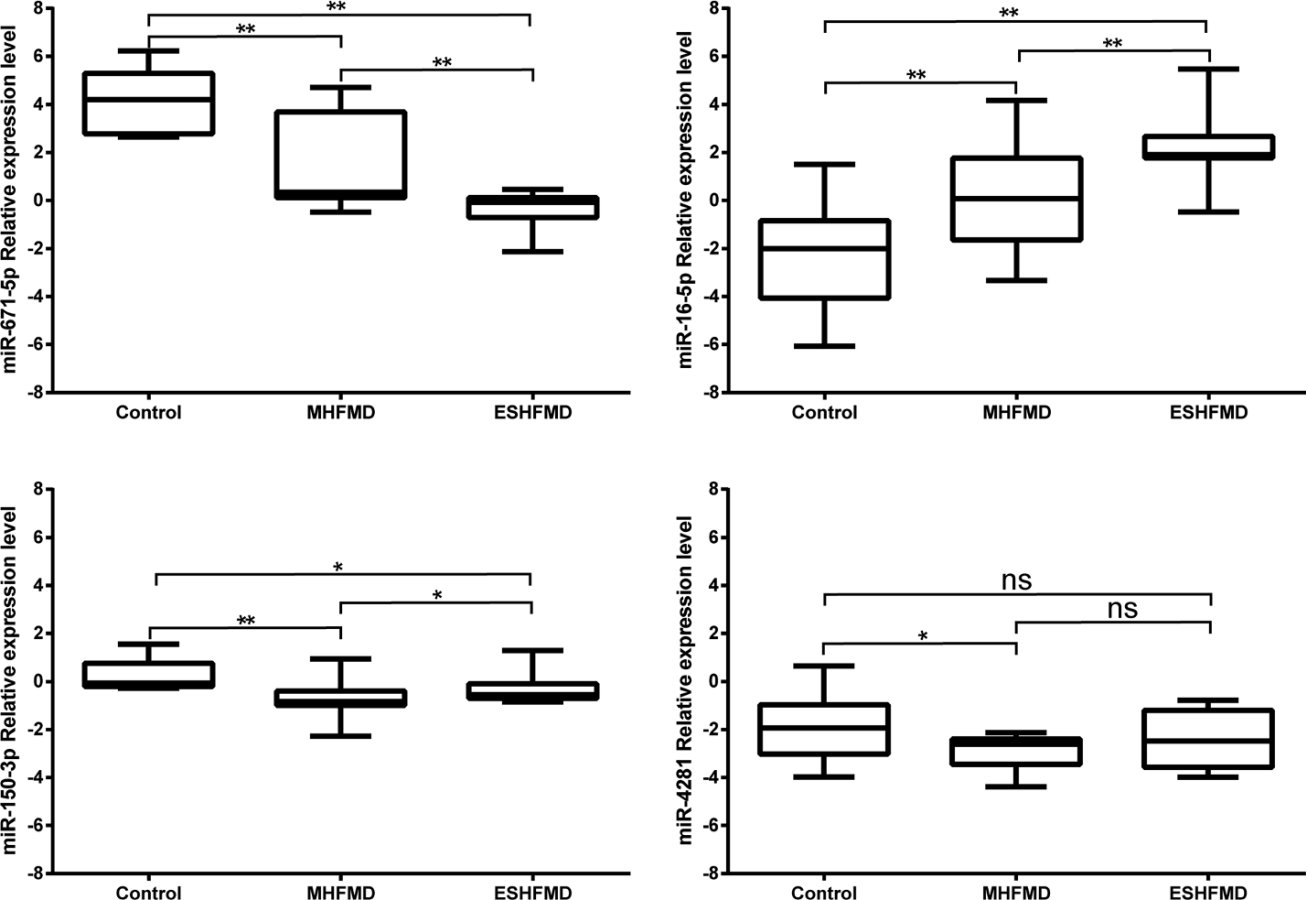
**The box plots depict the relative expression level of four miRNAs (miR-671-5p, miR-150-3p, miR-16-5p and miR-4281) assessed by qRT-PCR in ESHFMD, MHFMD, and control serum samples.** Statistically significant differences were determined using unpaired Student’s *t*-test.

The most differentially expressed miRNAs miR-671-5p, miR-16-5p, miR-150-3p, and miR-4281 (results from microarray analysis) were selected and tested using an independent cohort of 54 exosome samples (18 ESHFMD, 18 MHFMD, and 18 healthy control) subjected to qRT-PCR; all the miRNAs passed the quality control. miR-642a-3p expression was proposed as the normalization control for exosomal miRNA levels, as the expression level of miR-642a-3p was almost identical among control, MHFMD, and ESHFMD groups with almost no differences in raw Ct values, which was consistent with the microarray analysis results.Result indicated that the miR-671-5p, miR-16-5p, and miR-150-3p expression levels were significantly different between MHFMD or ESHFMD and the control; moreover, miR-671-5p was almost undetectable in ESHFMD in contrast to MHFMD and the control. miR-16-5p, miR-150-3p, and miR-671-5p showed the same changes as in the microarray analysis. It obtained that the miR-16-5p expression in exosome in HFMD serum samples were found especially higher than that in the normal children. In contrast, miR-671-5p and miR-150-3p levels were lower in HFMD than in controls. The serum miR-16-5p level increased by 5.98 and 10.31 fold in MHFMD and ESHFMD patients, respectively. Whereas, miR-4281 showed no significant differences between the ESHFMD group and the control group, or between the ESHFMD group and MHFMD group (Figure 3).

### Evaluation of miR-671-5p, miR-16-5p, and miR-150-3p as potential diagnostic markers

**Figure 4:**
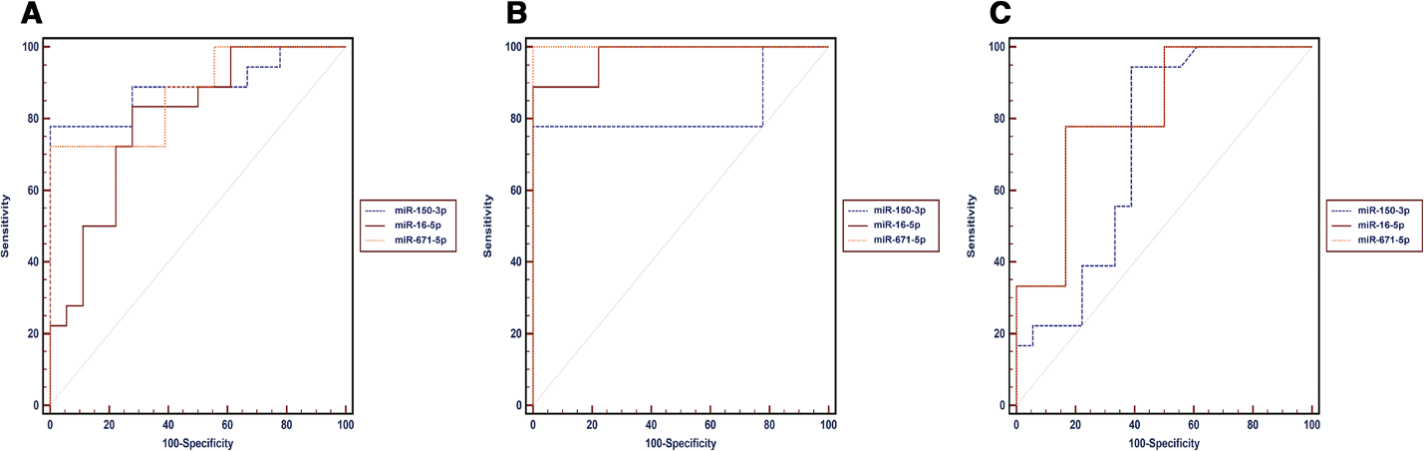
**Receiver operating characteristic (ROC) curve analysis of miR-671-5p, miR-150-3p, miR-16-5p was performed to discriminate MHFMD from the control (A), ESHFMD from the control (B), and MHFMD from ESHFMD (C).**

To determine whether serum miRNA levels in exsome can be used to distinguish patients with ESHFMD from those with MHFMD or controls, we established ROC curves to analyse the difference in miR-671-5p, miR-16-5p, and miR-150-3p serum levels between groups. Comparing the MHFMD and control groups, the ROC curve areas for miR-671-5p, miR-16-5p, and miR-150-3p were found to be 0.79 (95% CI, 0.62–0.91), 0.80 (95% CI, 0.63–0.91), and 0.89 (95% CI, 0.74–0.97), respectively. The specificity and the sensitivity of each of these miRNAs were 72% and 82%, 72% and 83%, and 100% and 78%, for the MHFMD and control groups (Figure 4A), respectively. These results clearly show that miR-671-5p, miR-16-5p, and miR-150-3p serum levels can distinguish MHFMD from healthy controls.We next compared the serum levels of these miRNAs between ESHFMD and control groups. The ROC curve areas of miR-671-5p, miR-16-5p, and miR-150-3p were 1.00 (95% CI, 0.90–1.00), 0.98 (95% CI, 0.86–1.00), and 0.83 (95% CI, 0.67–0.93), respectively. The specificity and the sensitivity for these miRNAs were 100% and 100%, 100% and 89%, and 100% and 78%, respectively, in the ESHFMD and control groups (Figure 4B). These results also demonstrate that the levels of these three miRNAs (miR-671-5p, miR-16-5p, and miR-150-3p) can distinguish ESHFMD from healthy controls.The comparison of ESHFMD with MHFMD indicated that miR-671-5p, miR-16-5p, and miR-150-3p levels are useful markers for discriminating patients with ESHFMD from those with MHFMD because the ROC curve area of miR-671-5p, miR-16-5p, and miR-150-3p was 0.82 (95% CI, 0.65–0.92), 0.76 (95% CI, 0.59–0.88), and 0.76 (95% CI, 0.58–0.88) and the specificity and the sensitivity were 83% and 78%, 78% and 89%, and 78% and 88% respectively, in the two groups (Figure 4C). Together, these results demonstrate that the miR-671-5p, miR-16-5p, and miR-150-3p serum levels can be used to distinguish MHFMD, ESHFMD, and control samples and reflected strong separation among these samples.

### GO Terms and KEGG pathway annotation of miRNA targets

**Figure 5:**
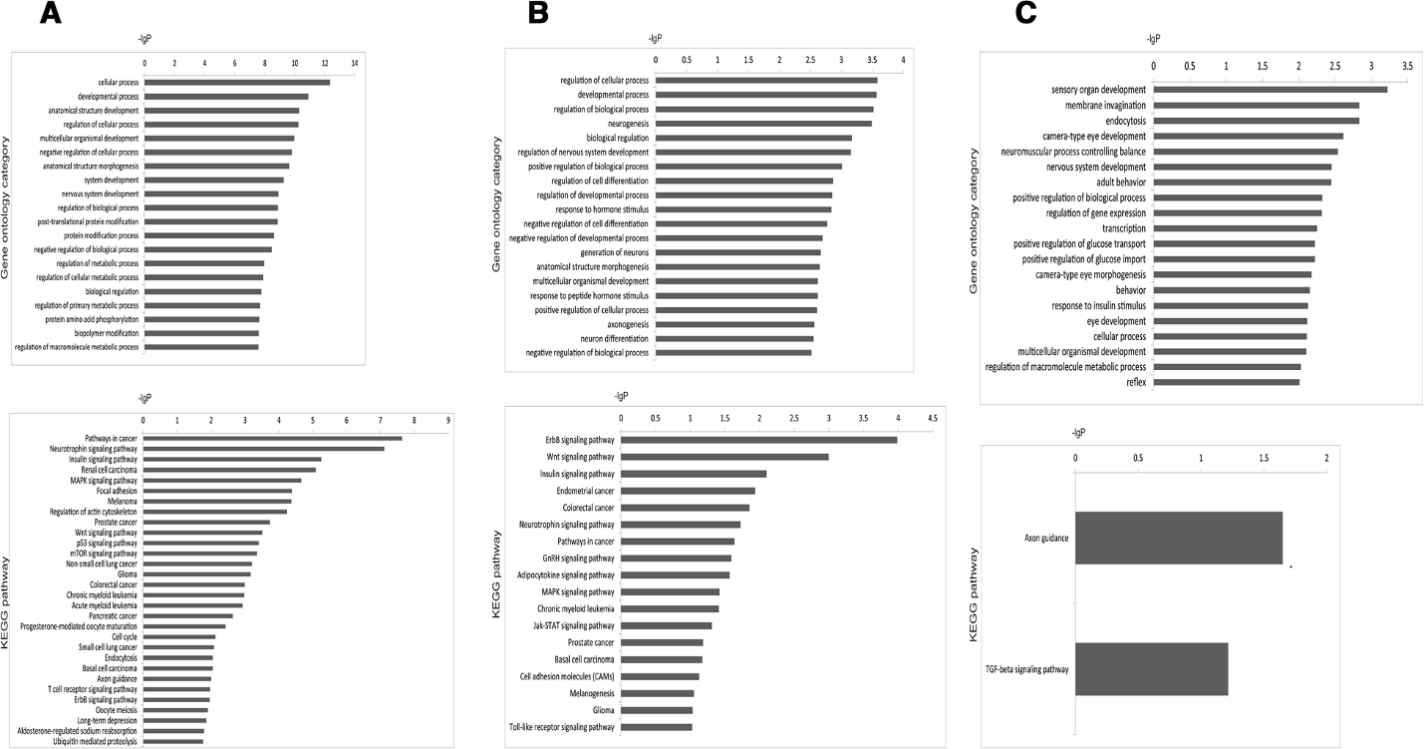
**GO category for putative target genes.** *P* < 0.05 was used as a threshold to select significant GO categories and for KEGG pathway analysis for putative target genes. *P* < 0.05 was used as a threshold to select significant KEGG pathways; lgP is the negative logarithm of the P-value. **(A)** miR-16-5p, **(B)** miR-150-3p, and **(C)** miR-671-5p.

GO biology process and KEGG pathway enrichments were performed by mapping the predicted target genes, and 20 biology processes for each miRNA and 30, 18, and 2 KEGG pathways for miR-16-5p, miR-150-3p, and miR-671-5p were annotated.The main GO categories annotation showed that developmental process and regulation of cellular process for the putative target genes of miR-16-5p and miR-150-3p, and neurogenesis, regulation of nervous system development, neuromuscular process controlling balance and nervous system development for the putative target genes of miR-150-3p and miR-671-5p were the most significantly enriched GO terms (Figure 5).The KEGG pathway enrichment analysis indicated that the putative targets for these miRNAs were mainly involved in pathways such as those related pathways in neurotrophin signalling pathway, insulin signalling pathway, TGF-beta signalling pathway, and MAPK signalling pathway (Figure 5).

## Discussion

Early and rapid separation of different disease infection condition may benefit controlling and prognosis prediction. Against HFMD, many substantial progresses in understanding the biology and pathogenesis agents continues [6, 7, 30]. As ESHFMD usually caused majority death, we need to recognize MHFMD from ESHFMD on the way to reduce mortality. Early clinical diagnosis, such as disease-associated miRNAs in exosome could serve as biomarkers, has the ability for understanding different infection states for HFMD. It could be also helpful for revealing some intriguing aspects regarding the potential function of these miRNAs in HFMD.

In this study, we investigated the expression levels of miRNA profile in exosome from serum samples in MHFMD and ESHFMD and healthy children. By the two-step screening and confirmation approach, we identified 3 miRNAs (miR-671-5p, miR-16-5p, and miR-150-3p) which were significantly different in patients in comparison to controls. To evaluate the efficiency of these miRNAs for diagnosing, ROC curves were constructed for each miRNA. Expression levels of three miRNAs (miR-671-5p, miR-16-5p, and miR-150-3p) showed good ability to efficiently distinguish MHFMD or ESHFMD from healthy control, with AUC that ranged from 0.79 to 1.00. Combination of three selected exsome miRNAs also created significantly increased the diagnosis efficiency to distinguish ESHFMD from MHFMD with AUC of 0.76 to 0.82. Furthermore, the miRNAs (miR-671-5p) was almost undetectable in ESHFMD when distinguishing it from MHFMD.

Moreover, the miR-16-5p expression in exosome was found especially higher, and miR-671-5p and miR-150-3p levels in exosome were particularly lower than that in healthy children. Recent publications revealed that the miR-16-5p is up-regulated in various human diseases including Alzheimer’s disease and prion disease. It has been identified as an important intercellular messenger mediating amyloid precursor protein (APP) protein formation [31]. Up-regulation of miR-16-5p during the early disease stage and decreased expression with disease progression was also found in prion disease [31]. It would therefore be interesting to determine that the miR-16-5p may have a neuroprotective role. Moreover, the miR-150-3p levels may be negatively correlated with plasma TNF-α level in patients [32] and those miR-671-5p levels may regulate gene expression to promote tumour growth [33]. From all the related study above, it may suggest that miR-16-5p, miR-150-3p and miR-671-5p in exosomes may play an important role in the movement of HFMD.

Further investigate the possible functions of miRNAs through GO terms and KEGG pathway annotation. The putative targets for these miRNAs were mainly involved in such as MAPK signalling pathway related to potential antiviral mechanisms [34], and the neurotrophin signalling pathway considered to influence the expression of APP [35]. The predicted target gene of miR-16-5p was CLU that could clear of beta amyloid peptide, which was one of the major brain lesions with Alzheimer’s disease [36–38]. Moreover, the polymorphic genes associated with Alzheimer’s disease delineate a clear pathway in young populations [39].

## Conclusions

Whereas definitive diagnosis depends on organismspecific detection results, our data recommended that exsomal miRNA profile provide a supplemental biomarker for differential infection stage at an early stage. They may share to several signalling pathways, for instance, the MAPK signalling pathway and neurotrophin signalling pathway, influenced by such as APP formation protein expression. Further studies, including the functional exploration of exosomal miRNA profile, will be needed to make these hypotheses served as the clinical diagnosis biomarkers.

## Authors’ original submitted files for images

Below are the links to the authors’ original submitted files for images. Authors’ original file for figure 1 Authors’ original file for figure 2 Authors’ original file for figure 3 Authors’ original file for figure 4 Authors’ original file for figure 5
