# Supplementary figures and images for: MicroRNA expression profile in exosome discriminates extremely severe infections from mild infections for hand, foot and mouth disease
Source: BMC Infect Dis. 2014 Sep 17;14:506. doi: 10.1186/1471-2334-14-506 (PMC4262082; doi:10.1186/1471-2334-14-506)

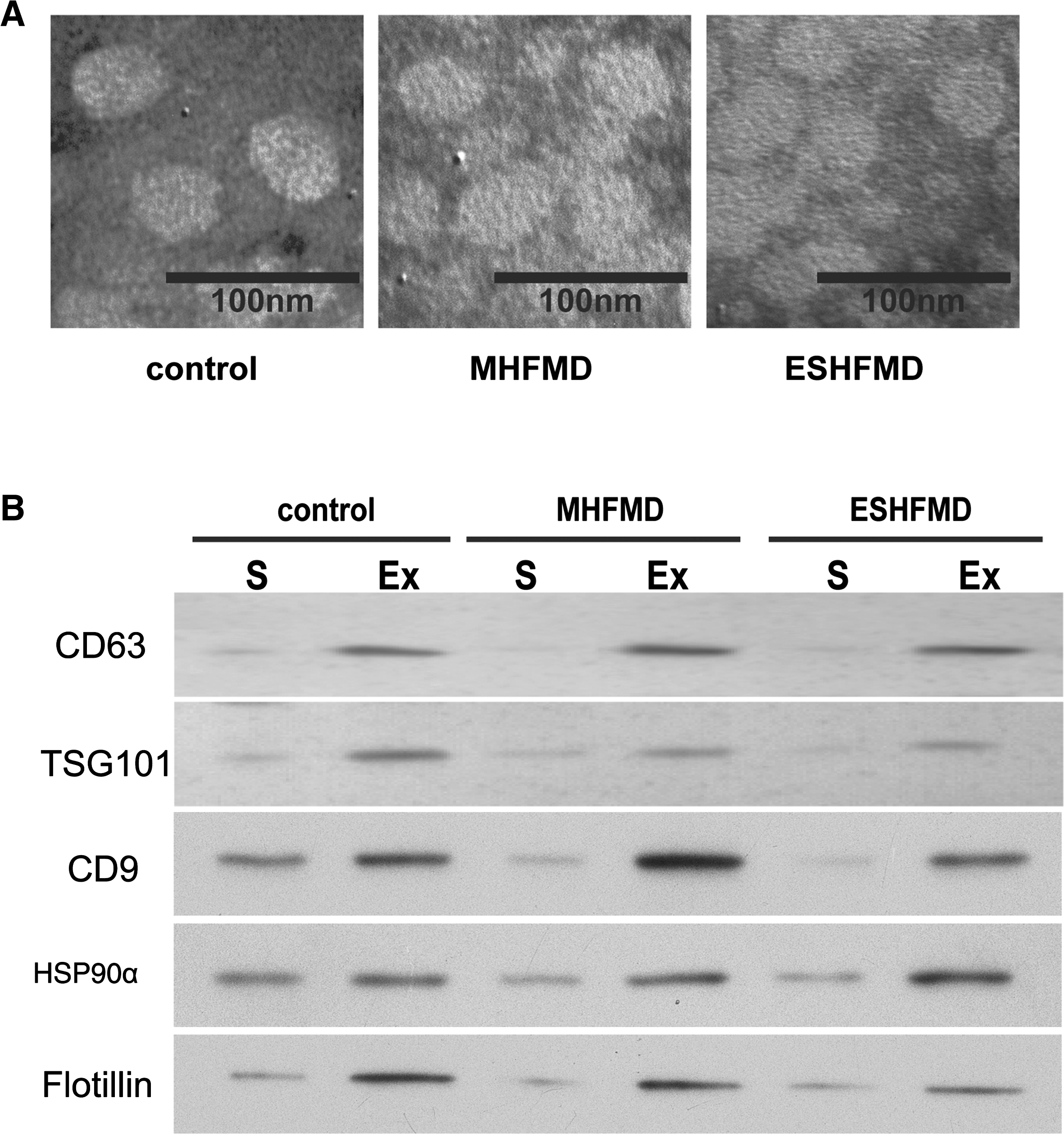

Supplement: Supplementary file 1 — Authors’ original file for figure 1 [file 12879_2014_3838_MOESM1_ESM.tif]

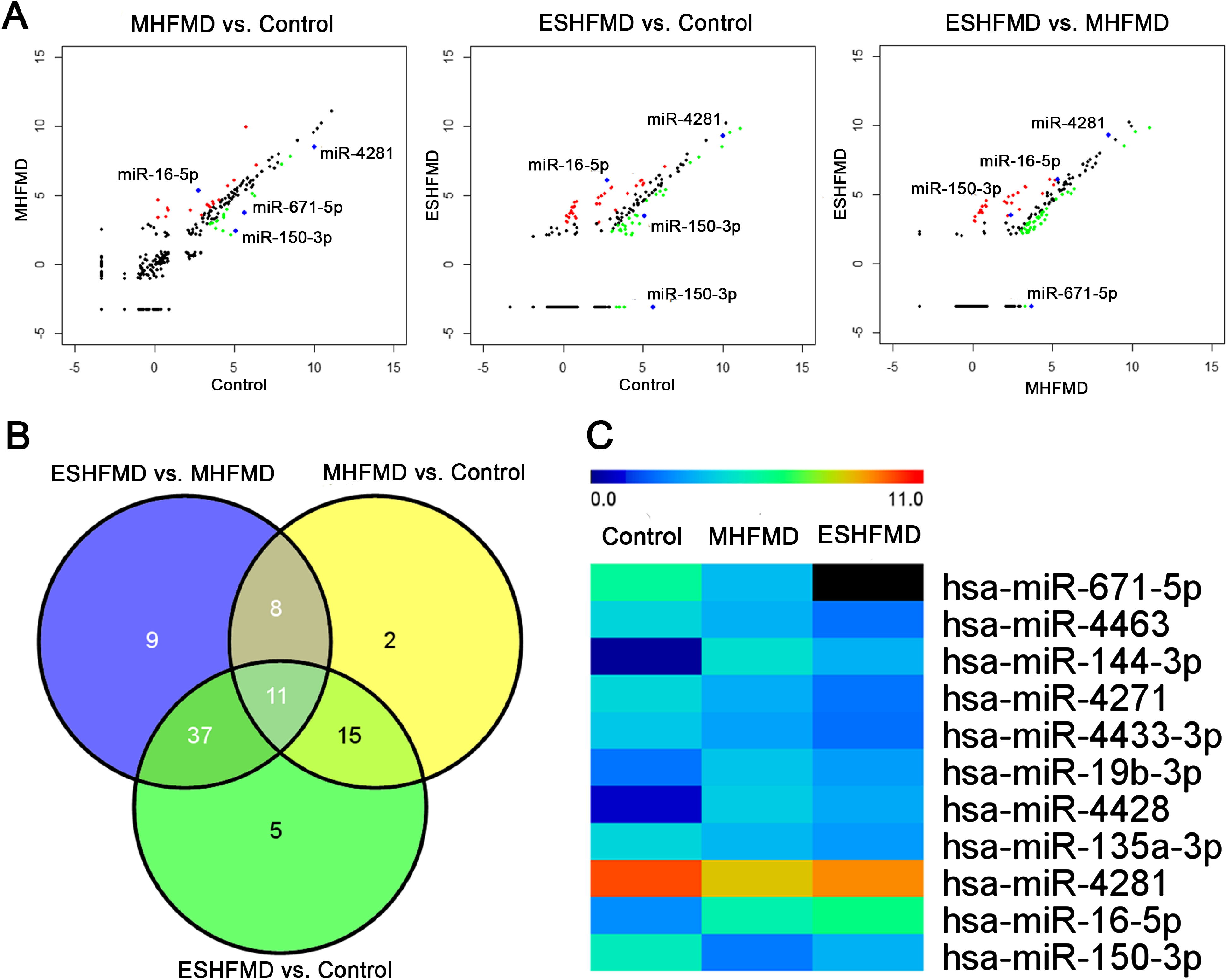

Supplement: Supplementary file 2 — Authors’ original file for figure 2 [file 12879_2014_3838_MOESM2_ESM.tiff]

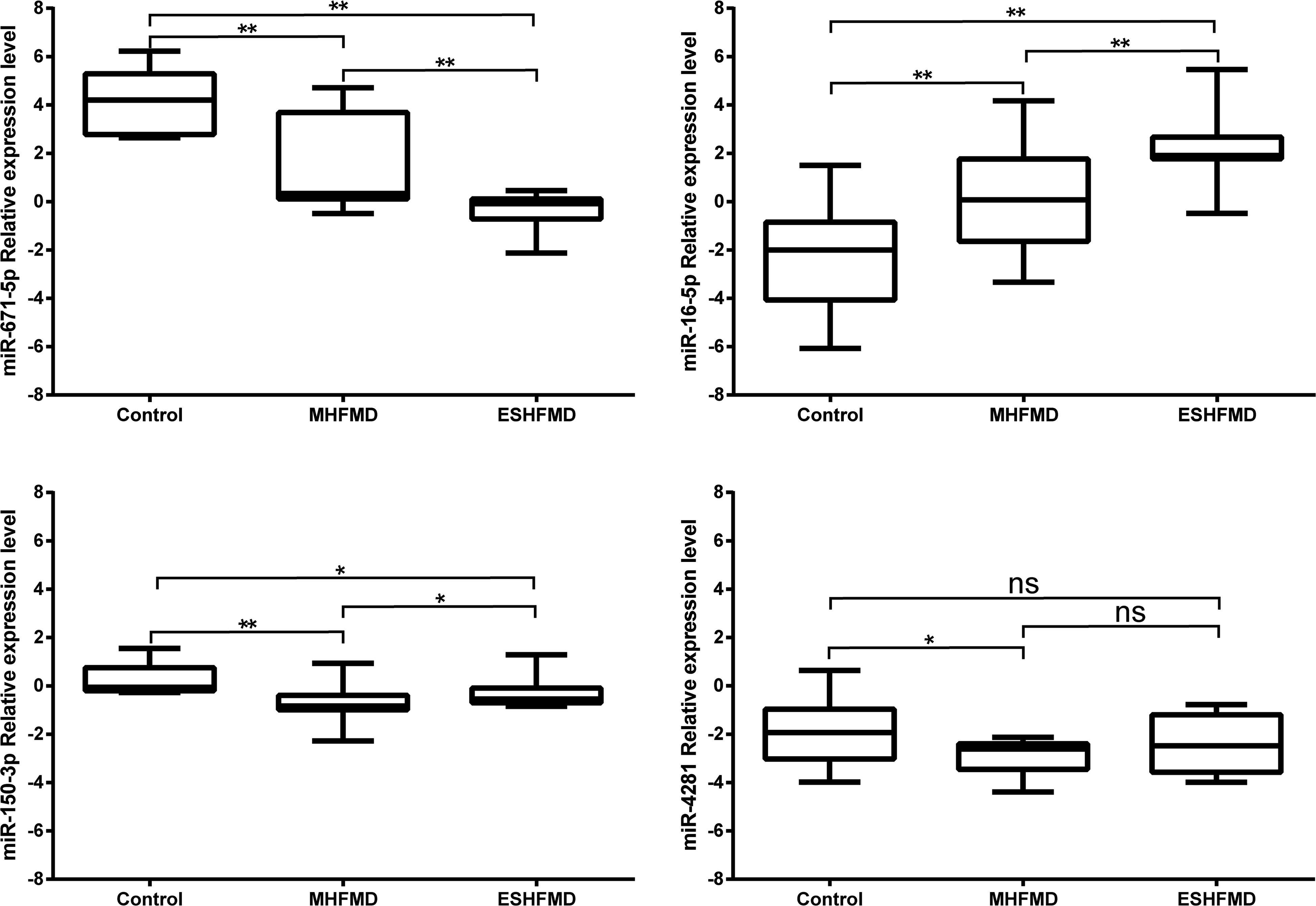

Supplement: Supplementary file 3 — Authors’ original file for figure 3 [file 12879_2014_3838_MOESM3_ESM.tif]

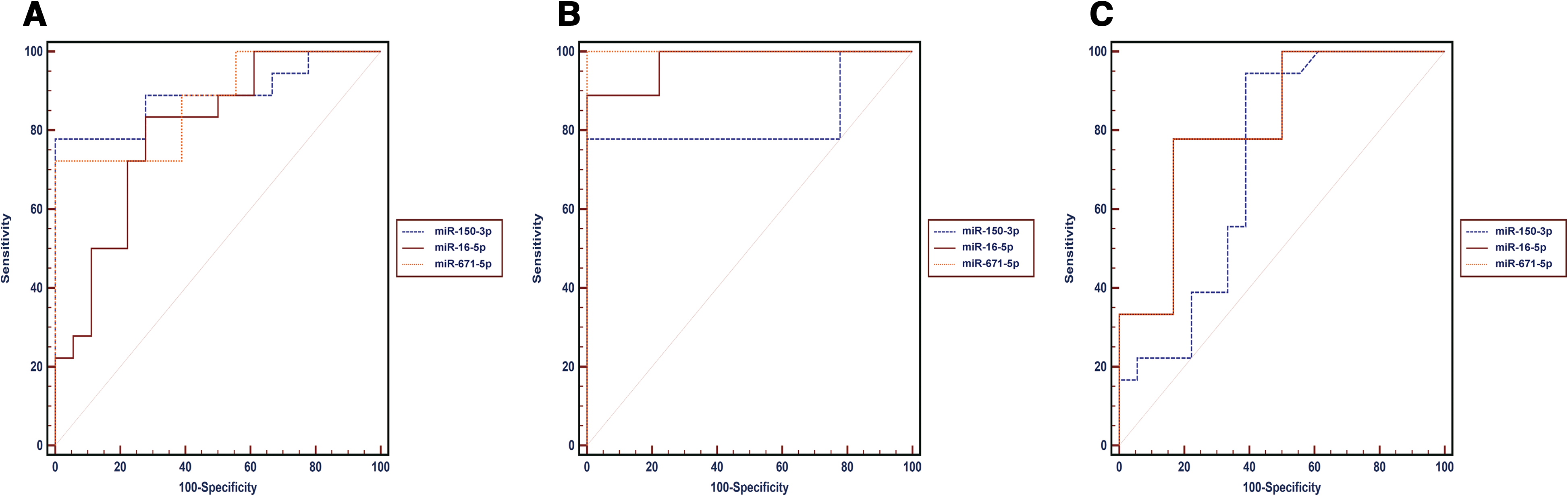

Supplement: Supplementary file 4 — Authors’ original file for figure 4 [file 12879_2014_3838_MOESM4_ESM.tif]

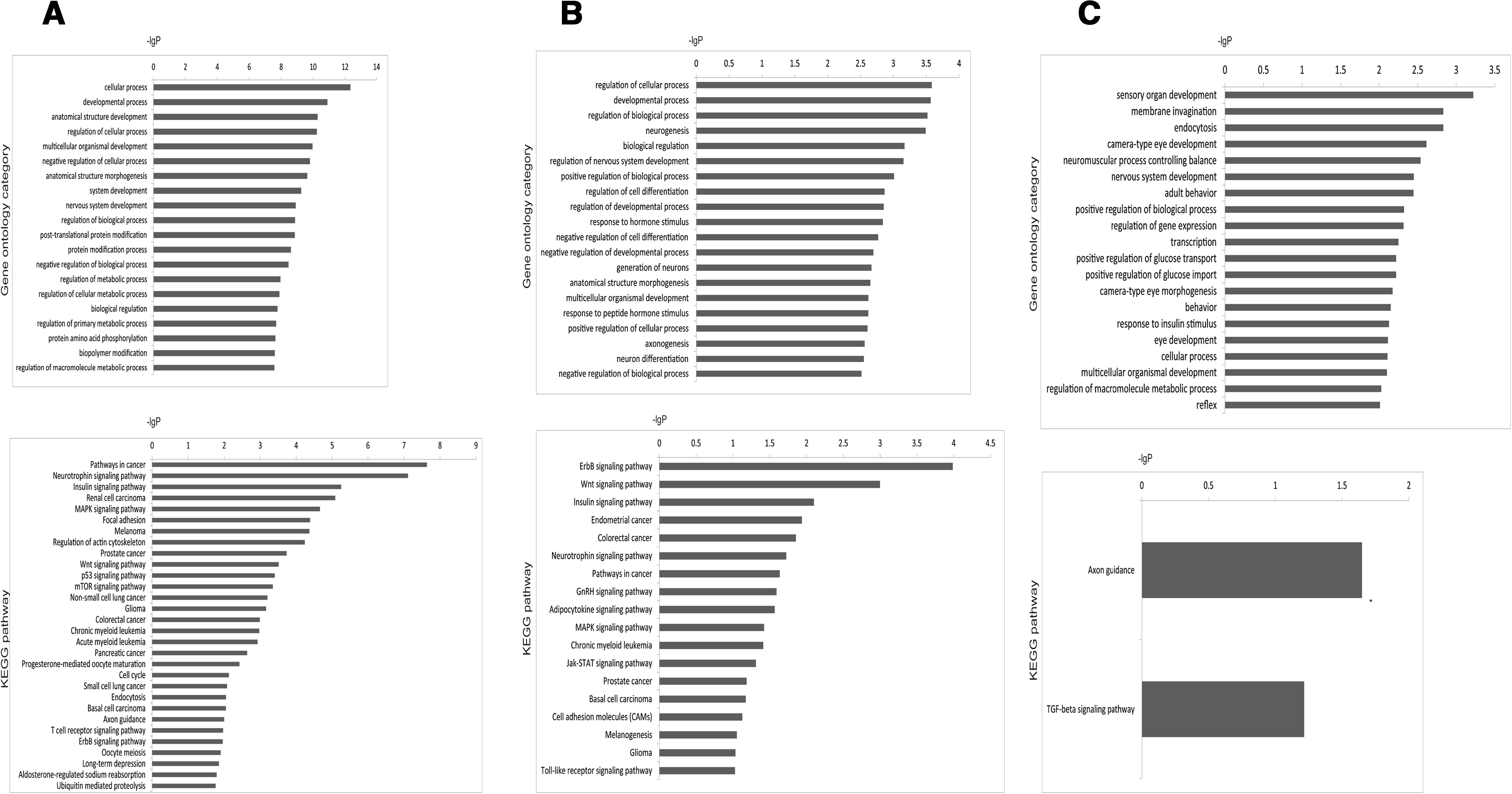

Supplement: Supplementary file 5 — Authors’ original file for figure 5 [file 12879_2014_3838_MOESM5_ESM.tif]
